# Microscopic simulation of free riding speed dynamics in bicycle traffic: Modeling heterogeneous context-dependent effects

**DOI:** 10.1371/journal.pone.0351469

**Published:** 2026-06-26

**Authors:** Guillermo Pérez Castro, Danil Belikhov, Fredrik Johansson, Heather Kaths, Johan Olstam

**Affiliations:** 1 The Swedish National Road and Transport Research Institute, Linköping, Sweden; 2 Division of Communications and Transport Systems, Linköping University, Norrköping, Sweden; 3 Chair of Bicycle Traffic, Center for Mobility and Transport, University of Wuppertal, Wuppertal, Germany; Khalifa University, UNITED ARAB EMIRATES

## Abstract

Simulation is a valuable tool for traffic planning that requires reliable modeling of traffic dynamics. Free flow speeds in bicycle traffic depend on the characteristics and preferences of the bicyclists, infrastructure design, and environmental conditions. However, existing models are limited in capturing changes in the speed during free riding, thereby reducing their applicability in bicycle traffic analysis. This study advances microscopic bicycle traffic simulation by developing and evaluating simulation models for free riding dynamics, aiming to capture the heterogeneous and context-dependent effects of infrastructure design (slopes, curves, and presence of intersections) and wind on bicyclist behavior. We implement three models within SUMO —(1) context-based speed distributions, (2) a speed regression model, and (3) a physics-based speed model derived from power output— and benchmark them against built-in models. All models are evaluated using empirical trajectory data from 57 bicycle commuters in semi-controlled experiments in Sweden and Germany. Results demonstrate that the proposed models outperform the existing baselines in replicating speed patterns. In this regard, the physics-based model provides the closest alignment to observed speeds. Omitting context dependency in free riding can result in substantially larger errors in speeds on uphills and downhills, and in deceleration on curves or when crossing intersections. Context-sensitive models enhance the accuracy of bicycle traffic simulation, thereby increasing their usefulness in planning and evaluating bicycling facilities that accommodate the diverse preferences of bicyclists.

## 1. Introduction

Microscopic traffic simulation supports the planning of well-functioning traffic systems through detailed analysis and performance evaluation of traffic flow. However, the usefulness of these analyses relies on the ability to replicate individual realistic movement and interactions within the traffic system, including free riding behavior. Free riding refers to movement that is not influenced by other road users or traffic control measures, but shaped by individual characteristics, preferences, and contextual factors such as infrastructure design and environmental conditions [1,2]. Unlike motorized traffic, bicycle speeds are highly sensitive to the environmental context, such as topography, infrastructure design, or weather, producing large speed variations within the bicyclist population, which in turn shape when and how bicyclists interact with other bicyclists through, e.g., overtaking and following. Studies of bicycle traffic flow characteristics, e.g., as in [3,4], provide valuable insights into how interactions between bicyclists shape fundamental traffic flow relationships, yet their representativeness is often limited to specific environmental contexts. Similarly, models describing these interactions in microscopic simulation are primarily adapted from motorized traffic [5,6], and thus also lack robust representation of this context dependency of speed choices.

A central challenge in modeling free riding is to accurately represent the speed choices, as this is vital for estimating travel times, diagnosing delays and discomfort [7,8], and safety risks [9], particularly those linked to infrastructure design or environmental conditions. Since bicycling is physically demanding and exposes users to weather and safety risks, the value of travel time savings for bicyclists is notably high [10]. Small reductions in travel time may substantially affect the perceived attractiveness of bicycling. Accurate simulation of speed changes under free riding directly enables evidence-based planning for attractive bicycle infrastructure. For example, microscopic traffic models can support traffic performance assessments by simulating downhill approach speeds at intersections, or optimize parking garage entrance and bridge designs by capturing diverse bicyclist responses to elevation. Additionally, precise speed modeling can enhance the estimation of travel time and energy expenditure for bicycle route planning.

Individual differences may account for up to 56% of the within‑population speed variance for a single trip [11]. When contextual trip features (topography, curvature, intersections, and wind) were considered, approximately 85% of the speed variance was explained. A similar decomposition of speed variation in [12] yielded 30% between individuals, 49% within a single trip, and 21% across trips by the same bicyclist. The type of bicycle is a major source of speed variation among bicyclists, as shown in several studies [2,13–17]. As discussed in [18,19], the pronounced heterogeneity in speed directly impacts traffic flow dynamics, necessitating accurate representation in microscopic simulation models.

Desired and/or maximum speed distributions are commonly used in microscopic simulation to represent speed preferences under free-flow conditions. For instance, speed limits generally determine free-flow speeds in motorized traffic, albeit with some variations. These distributions serve as input to simulate free speed dynamics while considering reactions to infrastructure elements. Given that most existing models for bicycle traffic are adaptations of frameworks originally developed for motorized vehicles, the influence of trip features on bicycle speeds is often oversimplified. Examples include the simulation approach implemented in PTV Vissim [20], where the maximum acceleration is modified as a function of the longitudinal slope (gradients). Similarly, the modified Krauss car-following model implemented in the Simulation of Urban MObility (SUMO) traffic simulation software [21,22] changes acceleration based on gradient. Both examples focus solely on longitudinal gradients and have been shown to underestimate gradient effects on bicycle speeds [23,24]. Other simulation approaches include context-specific speed distributions, as suggested, e.g., in [25], where separate desired speed distributions are assigned to varying infrastructure (e.g., uphills, downhills, curves), though limiting the ability to evaluate infrastructure design changes, as the desired speed must be specified by the analyst. While microscopic models may incorporate infrastructure influences, they typically overlook environmental impacts, including wind, heat, precipitation, and road surface conditions.

A promising alternative for simulating free riding centers on modeling speed as the outcome of trade-offs between travel time and energy expenditure (physical or perceived effort), as demonstrated in [26]. Power output is a reliable indicator for estimating energy expenditure in bicycling [27,28], making it an objective, quantifiable, and practical indicator of effort, as well as the primary input for simulation. Similarly to speed, empirical evidence shows that power output varies substantially both between bicyclists and in response to contextual features [11,23]. A key difference in a power-based simulation is that it uses a distribution of desired powers as input instead of desired speed distributions [23]. A desired power represents the effort each bicyclist is willing to exert to maintain a comfortable speed. By anchoring the simulation in the physics of human-powered motion, power-based models are an alternative for capturing the adaptive nature of bicycling behavior.

To better support bicycle traffic planning and infrastructure design through accurate simulation, we develop and evaluate three microscopic modeling approaches for free riding speed dynamics. The objective is to develop models that capture both the heterogeneity among bicyclists and the dependency on contextual and trip-related features, and evaluate their precision by comparing simulated speeds against observed trajectory data. All simulation models are implemented in SUMO, and are outlined as follows: (1) context-based speed distributions that facilitate the assessment of gradient effects; (2) regression model for speed that accounts for multiple trip features, and (3) a physics-based regression model that estimates speed based on kinetic energy dynamics derived from utilized power. We additionally compare the proposed models with two existing baseline models in SUMO: the default Krauss model and the gradient-adjusted variant KraussPS, mentioned previously.

## 2. Literature review

### 2.1 Speed modeling

Empirical studies —predominantly large-scale GPS analyses— have aimed to model the influence of bicyclists’ characteristics (age, type of bicycle, etc.), infrastructure design, and weather on bicyclist speed. These speed models can be categorized by their spatial resolution and intended application. Several of these studies have focused on segment-level speed predictions, e.g., as in [29–33], which limits their applicability in microscopic simulation, where higher-resolution (point-level) speed predictions are needed. Moreover, such models frequently rely on GPS data that often requires extensive pre-processing [34,35], and generally determine average population effects, limiting their applicability for capturing inter-individual variability. Since GPS tracking alone provides no information about the bicyclist’s surroundings, it also becomes challenging to isolate segments of free riding from those influenced by interactions with other road users.

Among point-level approaches, most studies rely on GPS-based regression techniques that relate speed to infrastructure features, though the comprehensiveness of explanatory variables varies significantly. For example, speeds were analyzed in [11] using 1 Hz-resolution trajectory data and found a notable variation in the impact of hills between individuals. The study also modeled reductions in speed on curves and at intersections, and revealed that the influence of wind on speed is linear: headwinds lead to a decrease in speed, whereas tailwinds cause an increase. Additional weather variables were examined in [12] such as temperature, rain, and humidity as determinants in speed choices. Other studies focus primarily on modeling the effects of topography, including [36–38]. Together, these findings highlight that bicyclist speed choices are highly context-dependent and shaped by both infrastructure features and environmental conditions. However, most existing models do not explicitly distinguish between free riding and traffic-constrained speeds; except for the work in [11].

Parkin and Rotheram [38] used ordinary least squares (OLS) regression to estimate population-average effects from gradients, reporting a relatively low adjusted R-squared of 0.266. More recent research has adopted multilevel mixed-effects frameworks [11,12,37], which can explicitly model individual-level heterogeneity and trip-level variation. These models include a broader set of trip features and have demonstrated improved model fit compared to OLS. The forward Markov model introduced in [36] incorporates dependence between subsequent observations and accounts for variation in bicyclists’ ability to plan ahead, explicitly modeling behavior in response to curvature and gradients. The Markov model outperformed generalized linear models (GLMs), highlighting the importance of modeling the dependence between observations in bicyclist behavior. Nonetheless, it struggled to capture high-speed values accurately, leading to reduced performance for fast bicyclists, with an average root mean square error (RMSE) in speed of approximately 1.97 m/s.

### 2.2 Acceleration modeling

Mathematical models have been developed based on empirical data to characterize bicyclist acceleration behavior, e.g., as in [1,39], including acceleration from standstill, deceleration to a full stop, and acceleration during cruising. Both studies found that polynomial functions best fit the observed acceleration profiles, though they noted that simpler models may also provide adequate approximations. However, neither study incorporates trip features such as gradients or curves. Moreover, Karakaya et al. [40] used a large-scale GPS dataset to empirically derive distributions for acceleration and deceleration, which were then used to calibrate acceleration parameters in SUMO. This work was extended in [41] by creating a new bicycle dynamics model for SUMO based on the same dataset.

A limited number of studies have directly examined how infrastructure —namely topography— affects acceleration. For instance, Parkin and Rotheram [38] developed an OLS model to estimate acceleration as a function of gradient, reporting a relatively low adjusted R-squared of 0.146. Pérez Castro et al. [42] used cross-sectional speed data to calibrate the maximum acceleration function implemented in PTV Vissim, specifically adjusting the gradient-acceleration relationship.

### 2.3 Power modeling

The correlation between power and energy expenditure in bicycling is strongly linear: as power increases, so does oxygen uptake and total energy expenditure [27,43]. Both personal and contextual factors influence the energy required to maintain a given speed, resulting in variability in energy expenditure across bicyclists. Given the close physiological relationship, power-based (also known as energy-based) models provide a promising framework for simulating bicyclist behavior.

Power-based models are grounded in the physics of bicycle dynamics, describing bicycling as a balance between the propulsive effort and resistive forces such as aerodynamic drag, rolling resistance, and gravity, e.g., as formalized in [44,45]. Building on this framework, modeling approaches have been developed in [23,46] to simulate bicyclist behavior in response to gradients, though with different approaches. The model proposed by Pérez Castro et al. [23] relies on a linear relationship between power and gradient, calibrated individually for each bicyclist to reflect variability within the population, both in their desired power and their desire (or ability) to compensate for the gradient. In contrast, Rothhämel [46] assumed bicyclists maintain constant power on uphills and reduced power on downhills, reflecting typical coasting behavior. Subsequent research attributed variation in power to gender, topography, curvature, the presence of intersections, wind, and individual tactical decisions to cope with uphills, such as boosting speed or coasting before ascents [11]. Moreover, Bigazzi and Lindsey [26] introduced a utility-maximization framework in which bicyclists choose their speed by weighing travel time, power-based energy expenditure, and stability control. This model captures the behavioral trade-offs underlying speed choice, and becomes especially useful when applied to large trajectory datasets —–potentially featuring multiple trips per bicyclist—– as demonstrated in [47], enabling robust estimation of individual preferences and parameters.

### 2.4 Research gaps

Existing research on bicyclist speed largely relies on segment‑level models or population‑average effects, limiting their applicability in microscopic traffic simulation, where individual, high-resolution, representations are utilized to capture heterogeneous behavior. Although intra-individual variability has been addressed in a limited number of studies, explicit distinctions between free riding and constrained behavior, as well as the inclusion of environmental factors such as wind, remain uncommon.

Acceleration models primarily focus on speed changes associated with starting from standstill or approaching a stop. While gradient‑dependent formulations exist, they provide limited guidance on how acceleration evolves over a trip or is adapted to varying contexts. In common microscopic simulation models, calibrating acceleration is inherently challenging because it often influences multiple interacting behaviors, including reaching a desired speed, overtaking dynamics, and interactions with infrastructure. For instance, existing simulation tools such as SUMO and PTV Vissim rely on gradient‑based acceleration adjustments, which have been shown to underestimate infrastructure effects on bicyclist speeds and may introduce unintended changes in traffic dynamics on flat segments [42]. Power‑based formulations offer a physics‑grounded alternative, in which context‑dependent variations in effort can be translated more explicitly into speed changes. To address the representation of heterogeneous free riding behavior under varying contexts, we develop and evaluate three microscopic simulation models within a unified framework.

### 2.5 Interaction models for bicycle traffic

Microscopic bicycle traffic simulation requires not only models for unconstrained behavior, but also interaction models that govern how bicyclists respond to other road users. Several modeling approaches have been applied to bicycle interactions, each with different assumptions and data requirements [5,48]. Car-following models, such as the Krauss model implemented in SUMO [21,22], adapt motorized vehicle logic to bicycles and rely on a desired speed as their primary behavioral input. Lane-free and sub-lane models have often been used to represent the lateral positioning behavior of bicyclists, e.g., as in [3,6,49]. Social force models have also been applied to bicycle traffic, as in [50–52], due to its ability to simulate lateral and longitudinal interactions simultaneously in a continuous two-dimensional plane. Cell-based approaches, such as cellular automata, offer computational efficiency for large-scale simulations [53], but typically have limited flexibility to represent the heterogeneity characteristic of bicycle traffic.

Across commonly used modeling approaches for bicycle traffic simulation, free riding is typically governed by a desired speed and an acceleration parameter that controls how quickly a bicyclist reaches that speed when unconstrained. In most implementations, these parameters remain fixed throughout the simulation and do not adapt to contextual changes along the route.

## 3. Methods

In this section, we outline data collection, microscopic modeling and simulation algorithms, and evaluation metrics to assess simulation performance.

### 3.1 Data collection and processing

To develop and evaluate modeling approaches, we use trajectory data of free riding behavior collected through semi-controlled experiments with instrumented bicycles (IBs), conducted in Linköping, Sweden, and Wuppertal, Germany [11,54]. A total of 57 (28 in Linköping and 29 in Wuppertal) bicycle commuters completed a single circuit route on their own traditional (non-electric) bicycle, equipped with sensors that recorded GPS positions, speed, power, and wind speed (in the direction of travel), at a sample frequency of 1 Hz. Additional instrumentation included video and audio recorders, and an event button for participants to log instances of constrained riding due to other road users or traffic signals. Approximately 75% of the population are men, and the average total weight of a rider+bicycle unit is 101.5 kg in Linköping and 98.2 kg in Wuppertal.

Participants were recruited in Linköping between September 28 and October 9, 2023, and in Wuppertal between June 22 and July 14, 2023. All participants were adults (≥18 years old) and signed a written informed consent form. The study was approved by the Swedish Ethical Review Authority (reference 2023-04518-01) and the Ethics Committee of the University of Wuppertal (reference SK/AE 230605). Detailed descriptions of the experimental procedure are available in [11]. Additional information regarding the ethical, cultural, and scientific considerations specific to inclusivity in global research is included in the Supporting Information (S1 Checklist).

The routes included a mix of hills, curves, flat segments, and intersections. In Linköping, participants rode exclusively on separated bicycle paths for 5 km, with moderate inclines up to 6 percent. In Wuppertal, participants rode approximately 3 km on a road shared with motorized traffic, with steep inclines up to 10 percent. Participants were instructed to ride at their preferred pace —as in a typical commute— and to report if their behavior was influenced by other road users or traffic signals.

Elevation and gradient data were extracted from high-resolution ground elevation models [55,56], and synchronized with the GPS trajectories at 1-meter resolution. To ensure the analysis focused on free riding behavior, all trajectory segments where participants reported being influenced by other road users or signals were excluded. This filtering removed 3.6% of the data in Linköping and 8.9% in Wuppertal.

### 3.2 Modeling framework

To simulate free riding speed dynamics, we first define the concept of desired speeds and powers, and then describe the two core modeling frameworks utilized: bicycle dynamics modeling and mixed-effects linear regression.

#### 3.2.1 Desired speed and power.

In our dataset, we observe the desired speeds on straight, flat segments (gradients up to ±1%) with low wind (<3 m/s in magnitude), where the participants are uninfluenced by other road users or traffic signals, and are cruising at steady speed (acceleration within ±0.03 m/s^2^). The desired power is defined as the power measured under these conditions.

By extracting these values for each participant, we capture the heterogeneity present in the population coming from differences in age, gender, physical capabilities, and individual preferences. These distributions provide the basis for modeling inter-individual variability in microscopic traffic simulation.

#### 3.2.2 Bicycle dynamics.

A physics-based bicycle dynamics model balances the propulsive power output of a bicyclist against the total resistive forces encountered during motion. This approach is grounded in energy conservation principles and builds on the framework detailed in [44]. The model expresses the instantaneous acceleration as:


$$\begin{array}{c} \dot{v}(t)=\frac{1}{M}\left[\frac{\eta p(t)}{v(t)}-F(x(t),v(t))\right], \end{array}$$ (1)

where *p*(*t*) is the power at time *t*, *v*(*t*) is the speed of the bicyclist at time *t*, η is the chain efficiency, and *M* is the total effective mass including rotational inertia, calculated as *M* = *m* + *I*/*r*^2^, where *m* is the total mass of the bicycle–rider unit, *I* is the wheel inertia, and *r* is the wheel radius. The total resistive force, *F*, is the set of all resistances as:


$$\begin{array}{c} F(x(t),v(t))=\frac{1}{2}C_{d}A\rho (v(t)+w(t){)}^{2}+C_{rr}mg+\alpha_{0}+\alpha_{1}v(t)+mgs(x(t)). \end{array}$$ (2)

The terms in Eq. 2 correspond to aerodynamic drag, rolling resistance, bearing resistance, and gravity, where $C_{d}A$ is the drag area, *w* is the wind speed in the direction of travel (negative for tailwinds), ρ is air density, $C_{rr}$ is the rolling resistance coefficient, *g* is the gravitational acceleration, $\alpha_{0}$ and $\alpha_{1}$ are bearing friction coefficients, and *s*(*x*(*t*)) is the road longitudinal gradient based on the elevation profile, and is thus, dependent on the longitudinal position *x*(*t*) of the bicyclist.

To avoid numerical instability at *v*(*t*)=0, we replace *v*(*t*) by the kinetic energy defined as


$$\begin{array}{c} e(t)=\frac{1}{2}Mv(t{)}^{2}, \end{array}$$ (3)

and transform the physical model to instead solve for changes in kinetic energy as


$$\begin{array}{c} \dot{e}(t)=Mv(t)\dot{v}(t)=\eta p(t)-F\left(x(t),\sqrt{\frac{2e(t)}{M}}\right)\sqrt{\frac{2e(t)}{M}}, \end{array}$$ (4)

with all speed-dependent terms replaced by their kinetic energy equivalents.

In a population of bicyclists, resistance parameters ($C_{d}A$ and $C_{rr}$), may vary due to differences in the physical characteristics of rider-bicycle units and riding positions [57,58]. For each participant in the Linköping experiment, a series of calibration rides are performed to estimate personalized resistance parameters through coast-down tests. These tests measure the motion of bicyclists as they coast from cruising speed to a full stop without braking; this approach is only feasible where wind speed measurements are available. By reformulating the equations of bicycle dynamics, bicycle resistance parameters are calibrated by minimizing the difference between observed and predicted stopping distances (see S1 Appendix in the Supporting Information for the detailed implementation).

#### 3.2.3 Mixed-effects models.

To quantify the impact of both individual preferences and trip features on speed and power, we use a two-level mixed-effects regression modeling framework implemented via the MixedLM function [59]. Linear mixed-effects models are statistical models that incorporate both fixed and random effects, representing responses that are consistent across all participants (fixed effects), and individual-specific variations (random effects). The general model can be written as:


$$\begin{array}{c} y_{ij}=\beta_{0}+\sum_{k=1}^{p}\beta_{k}x_{ijk}+u_{0j}+\sum_{k=1}^{q}u_{kj}x_{ijk}+\epsilon_{ij}, \end{array}$$ (5)

where $y_{ij}$ is the outcome (speed or power) for observation along the trajectory *i* (level 1) of bicyclist *j* (level 2), $\beta_{0}$ and $\beta_{k}$ are fixed intercepts and slopes, $x_{ijk}$ are predictors (e.g., gradients, winds), *u*_0*j*_ and $u_{kj}$ are the random (individual) intercepts and slopes, and $\epsilon_{ij}$ is the residual error term, assumed to follow $𝒩(0,\sigma^{2})$.

A mixed-effects modeling framework allows us to disentangle population-level effects from individual-specific deviations, making it a suitable tool for describing heterogeneous free riding. We interpret the estimated intercepts *u*_0*j*_ as indicative of the desired speed and power, reflecting behavior under comfortable and undisturbed conditions, and parameters $u_{kj}$ as the desire (or ability) to compensate for a predictor $x_{ijk}$.

The models are constrained to trip characteristics as determinants of free riding, including predictors related to topography, curvature, the presence of intersections with both bicycle and motorized traffic, and wind speed. Given that free riding on a hill is not solely determined by its steepness at a given point, but also by its length and elevation profile, we disentangle the impact of topography by incorporating three predictors: the local longitudinal gradient that captures the immediate resistance or assistance experienced at each point along the route, the cumulative elevation gain or loss from the start of the incline, accounting for the total altitude change over a non-flat segment, and a binary indicator for downhill-to-uphill transitions. By modeling these aspects separately, we isolate the specific effect of local gradients from the influence of prolonged hills and tactical adjustments in anticipation of changing terrain. To account for anticipatory or tactical behavior, we include a variable “is uphill ahead” that is active only on downhills directly preceding an uphill; it captures the anticipatory increase in power (or speed) during the descent, which ranges from coasting to varying degrees of boosting across bicyclists.

Curvature is included as a measure of horizontal alignment, defined as the rate of directional change per unit distance (in m^−1^), where sharper curves correspond to higher values. The presence of intersections is represented as a binary variable defined for any location within 20 meters before or after an intersection. Headwinds and tailwinds have similar impacts on the speed and power but in the opposite direction [11]. Therefore, we include a single linear predictor for wind speed in the current model, with positive values for headwinds and negative for tailwinds.

Using the collected trajectory data, we independently estimate mixed-effects models for both speed and power at each location. For the power model, we use a hybrid power variable as the dependent outcome. When the bicyclist is actively pedaling, the measured power is used. When the power reading is zero (indicating coasting or braking), we replace it with a substitute power value estimated by solving the physical bicycle dynamics model using the observed speed profile. In this case, negative estimated power is interpreted as an indication of braking and replaces the zero reading to capture deceleration behavior in the simulation.

### 3.3 Simulation models and algorithms

We use SUMO to replicate the conditions of the experiments. The simulation environment is constructed using the network layout, edges, and junctions from OpenStreetMap. We implement time-discrete simulation algorithms, with a time step (Δt) of 0.1 seconds. At each simulation step, the state variables are updated using SUMO’s default Euler integration scheme, where the position of each bicyclist is advanced by assuming their speed remains constant during the time step. Simulation algorithms are implemented via the TraCI (Traffic Control Interface), which provides real-time control over the SUMO simulation [60].

To replicate and evaluate the free riding behavior observed for all participants, we simulate each individual trip separately, i.e., only one bicyclist is present per simulation run. This approach ensures that simulated speed dynamics can be directly compared to the observed trajectories, free from any interactions with other simulated entities. For each simulated bicyclist *j*, we specify a personalized desired and maximum speed, *v*_0,*j*_ and *v*_max,*j*_ respectively, both derived from empirical observations; the maximum speed value serves as an upper bound in the simulation.

At every simulation time step, we derive a targeted speed (*v*_target,*j*_) to be achieved in the next time step. The targeted speed is constrained by maximum acceleration or deceleration, the maximum speed, and must be nonnegative. If the targeted speed cannot be reached within these limits, it is adjusted to the nearest feasible value. All examined models share the same kinematic update (Algorithm 1), which enforces the acceleration, deceleration, and speed bounds. They differ only in how *v*_target,*j*_(*t*) is computed at each time step.

**Algorithm 1. Kinematic update.**

1: **procedure** UpdateState($v_{\text{target},j},v_{j}(t)$)

2:  **if** $v_{\text{target},j}\geq v_{j}(t)$ **then**

3:   $v_{j}(t+\Delta t)\leftarrow \min\left[v_{\text{target},j},v_{j}(t)+a_{\max}\Delta t,v_{\max,j}\right]$

4:  **else**

5:   $v_{j}(t+\Delta t)\leftarrow \max\left[v_{\text{target},j},v_{j}(t)-b_{\max}\Delta t,0\right]$

6:  **end if**

7:   $x_{j}(t+\Delta t)\leftarrow x_{j}(t)+v_{j}(t)\Delta t$ ▷ Euler integration

8:   **return** $v_{j}(t+\Delta t),x_{j}(t+\Delta t)$

9: **end procedure**

Elevation profiles, sampled every 1 meter, are incorporated from high-resolution ground elevation models [55,56]. During the simulation, we manually update key infrastructure features —including gradient, curvature, intersections, and wind speeds—– at each time step based on the current position of the simulated bicyclist. To focus on comparing the accuracy of the implemented models, we refrain from using built-in TraCI functions to get infrastructure data —such as gradients—– due to evidence showing these produce noisy gradient values that can negatively impact simulation output.

The following subsections describe the three proposed simulation models, and the two benchmark SUMO models used for comparison. The simulation code and model implementations are publicly available (see our Data Availability Statement).

#### 3.3.1 Context-based speed distributions.

Gradient is identified as a significant trip-context variable affecting bicycling speed [11]. This model extends the concept of gradient-specific speed distributions proposed in [25] by incorporating linear interpolation for smoothed speed transitions as gradients change. Based on the observed desired speed distribution on flat terrain, we assign each bicyclist a percentile that reflects their relative position within the range of preferred speeds in the population. This percentile remains constant throughout the simulation. At each time step, the targeted speed is computed from the empirical speed distributions corresponding to the current gradient. Since empirical speed observations are available for discrete gradient intervals, we apply linear interpolation between the percentile speed values from the two nearest gradient bins that bracket the current gradient (e.g., [–1%, 1%], [1%, 3%], etc.) to avoid abrupt or noisy shifts in acceleration. Algorithm 2 summarizes the procedure.

**Algorithm 2. Context-based speed distribution model**

**Require:** Empirical speed distributions $v_{j}^{S}(\pi_{j})$ for gradient bins S∈𝒮; bicyclist *j* with fixed percentile $\pi_{j}$ at flat terrain (*S*=0); simulation horizon *T*

**Ensure:** Simulated trajectory $\{(x_{j}(t),v_{j}(t)){\}}_{t=0}^{T}$

1: Initialize $x_{j}(0),\;v_{j}(0)$ from trajectory of bicyclist *j*

2: **for** t=0,Δt,2Δt,…,T−Δt **do**

3:   $\gamma \leftarrow s(x_{j}(t))$            ▷ Gradient at current position

4:   Select bins $S_{\text{lower}},S_{\text{upper}}\in 𝒮$ with $S_{\text{lower}}\leq \gamma <S_{\text{upper}}$

5:   $v_{\text{target},j}\leftarrow$ linear interpolation of $v_{j}^{S_{\text{lower}}}(\pi_{j})$ and $v_{j}^{S_{\text{upper}}}(\pi_{j})$ at γ

6:   $v_{j}(t+\Delta t),\;x_{j}(t+\Delta t)\leftarrow \mathrm{UpdateState}(v_{\text{target},j},\;v_{j}(t))$

7: **end for**

#### 3.3.2 Regression speed model.

We fit a mixed-effects linear regression model to empirical speed data to estimate the targeted speed as a function of local trip features, namely topography, curvature, presence of intersections, and wind speed in the direction of travel. Algorithm 3 summarizes the simulation procedure.

**Algorithm 3. Regression speed model**

**Require:** Bicyclist *j* with individual-specific random effects $u_{0j},u_{kj}$ and fixed effects $\beta_{0},\beta_{k}$ from the mixed-effects speed model; trip features $x_{ijk}$ (topography, curvature, intersections, and wind speed); simulation horizon *T*

**Ensure:** Simulated trajectory $\{(x_{j}(t),v_{j}(t)){\}}_{t=0}^{T}$

1: Initialize $x_{j}(0),\;v_{j}(0)$ from trajectory of bicyclist *j*

2: **for** t=0,Δt,2Δt,…,T−Δt **do**

3:   Retrieve local trip features $x_{ijk}$ at position $x_{j}(t)$

4:   *v*_target,*j*_ from Eq. 5

5:   $v_{j}(t+\Delta t),\;x_{j}(t+\Delta t)\leftarrow \mathrm{UpdateState}(v_{\text{target},j},\;v_{j}(t))$

6: **end for**

#### 3.3.3 Physics-based regression speed model.

We use a mixed-effects linear regression model to estimate power based on explanatory variables related to topography, curvature, intersections, and wind speed. The estimated power (*p*(*t*) is then used to compute the targeted speed using the physical model of bicycle dynamics. With an estimated power, we track the change in kinetic energy ${\dot{e}}_{j}(t)$ for each bicyclist during the simulation period, using Eqs. 2 and 4, which account for all relevant resistance forces (aerodynamic drag, rolling resistance, bearing friction, and gravity). Unlike traditional simulation models, this approach does not rely on a desired speed distribution as input. Instead, it is based on a distribution of desired power. To ensure physical realism, the simulated power is constrained by the maximum power value *p*_max,j_, empirically derived for each bicyclist, ensuring that power cannot exceed physical capabilities. Furthermore, we constrain the estimated power to be nonnegative to prevent stalling and difficulties in resuming motion. Algorithm 4 summarizes the procedure.

**Algorithm 4. Physics-based regression speed model**

**Require:** Bicyclist *j* with individual-specific random effects $u_{0j},u_{kj}$ and fixed effects $\beta_{0},\beta_{k}$ from the mixed-effects power model; maximum power $p_{\max,j}$; bicycle dynamic parameters $m,\rho ,C_{d}A,C_{rr},\alpha_{0},\alpha_{1},I,r,\eta ,g$ for bicyclist *j*; trip features $x_{ijk}$ (topography, curvature, intersections, and wind speed); simulation horizon *T*

**Ensure:** Simulated trajectory $\{(x_{j}(t),v_{j}(t)){\}}_{t=0}^{T}$

1: Initialize $x_{j}(0),\;v_{j}(0),\;e_{j}(0)$ from trajectory of bicyclist *j*

2: **for** t=0,Δt,2Δt,…,T−Δt **do**

3:   Retrieve local trip features $x_{ijk}$ at position $x_{j}(t)$

4:   $p_{j}(t)\leftarrow$ Eq. 5 subject to $0\leq p_{j}(t)\leq p_{\text{max,j}}$

5:   ${\dot{e}}_{j}(t)\leftarrow$ Eqs. 2 and 4

6:   $e_{j}(t+\Delta t)\leftarrow e_{j}(t)+{\dot{e}}_{j}(t)\;\Delta t$

7:   $v_{\text{target},j}\leftarrow \max\left[0,\;\sqrt{2\;e_{j}(t+\Delta t)/M}\right]$

8:   $\begin{array}{c} v_{j}(t+\Delta t),\;x_{j}(t+\Delta t)\leftarrow \mathrm{UpdateState}(v_{\text{target},j},\;v_{j}(t)) \end{array}$

9: **end for**

#### 3.3.4 SUMO benchmark.

We use two models available in SUMO as benchmarks: the default Krauss model and its gradient-adjusted variant, KraussPS. The standard Krauss model is the default behavioral model in SUMO, which does not explicitly consider effects from any trip features. It serves as a baseline for evaluating simulation output when context-dependency is not modeled. The Krauss model is a stochastic car-following model that updates the speed of a vehicle based on safe acceleration, maximum speed, and random fluctuations (dawdling) [21]. Under free riding conditions, the targeted speed is obtained by applying a constant acceleration until the desired speed is reached, as indicated in Algorithm 5.

**Algorithm 5. Krauss model (SUMO default)**

**Require:** Bicyclist *j* with desired speed *v*_0,*j*_; maximum acceleration $a_{\max}$; simulation horizon *T*

**Ensure:** Simulated trajectory $\{(x_{j}(t),v_{j}(t)){\}}_{t=0}^{T}$

1: Initialize $x_{j}(0),\;v_{j}(0)$ from trajectory of bicyclist *j*

2: **for** t=0,Δt,2Δt,…,T−Δt **do**

3:  **if** $v_{j}(t)<v_{0,j}$ **then**

4:   $a\leftarrow a_{\max}$

5:  **else**

6:   a←0

7:  **end if**

8:  $v_{\text{target},j}\leftarrow v_{j}(t)+a\;\Delta t$

9:  $v_{j}(t+\Delta t),\;x_{j}(t+\Delta t)\leftarrow \mathrm{UpdateState}(v_{\text{target},j},\;v_{j}(t))$

10: **end for**

The KraussPS variant extends the default model by incorporating the effect of longitudinal gradient on acceleration and maximum speed, as indicated in Algorithm 6.

**Algorithm 6. KraussPS model (gradient-adjusted variant)**

**Require:** Bicyclist *j* with desired speed *v*_0,*j*_; maximum acceleration $a_{\max}$ and deceleration $d_{\max}$; gravitational acceleration *g*; simulation horizon *T*

**Ensure:** Simulated trajectory $\{(x_{j}(t),v_{j}(t)){\}}_{t=0}^{T}$

1: Initialize $x_{j}(0),\;v_{j}(0)$ from trajectory of bicyclist *j*

2: **for** t=0,Δt,2Δt,…,T−Δt **do**

3:   Retrieve local gradient θ (in radians) at position $x_{j}(t)$

4:   $a_{\text{adj}}\leftarrow \max\left[0,\;a_{\max}-g\sin(\theta )\right]$

5:   $v_{\text{max-adj},j}\leftarrow \max\left[\sqrt{a_{\text{adj}}/a_{\max}}\cdot v_{0,j},\;v_{j}(t)-d_{\max}\;\Delta t\right]$

6:   $v_{\text{target},j}\leftarrow \max\left[a_{\max}/2,\;\min\left[v_{j}(t)+a_{\text{adj}}\;\Delta t,\;v_{\text{max-adj},j}\right]\right]$

7:   $v_{j}(t+\Delta t),\;x_{j}(t+\Delta t)\leftarrow \mathrm{UpdateState}(v_{\text{target},j},\;v_{j}(t))$

8: **end for**

### 3.4 Evaluation metrics

To evaluate each simulation model, we compare simulated outputs against empirical data of speed, acceleration, and power. For aggregate analysis, we calculate the mean speed, mean acceleration, and mean power at every 1-meter interval along the route by averaging values across all simulated trajectories within the population. This approach enables a broad assessment of how well each simulation reproduces spatial patterns observed along the route. To assess performance at the individual level, we calculate the root mean squared error (RMSE) of speed for each participant (trajectory). These individual errors are then summarized with population-level confidence intervals. Furthermore, we disaggregate RMSE values by gradients and route segment: the entire route, downhill-to-uphill transitions, curves, and intersections. The individual assessment not only defines the anticipated error margin for each simulation model but also facilitates an analysis by segment type, allowing for the identification of the advantages and drawbacks of each model.

## 4. Parametrization of models

**Fig 1:**
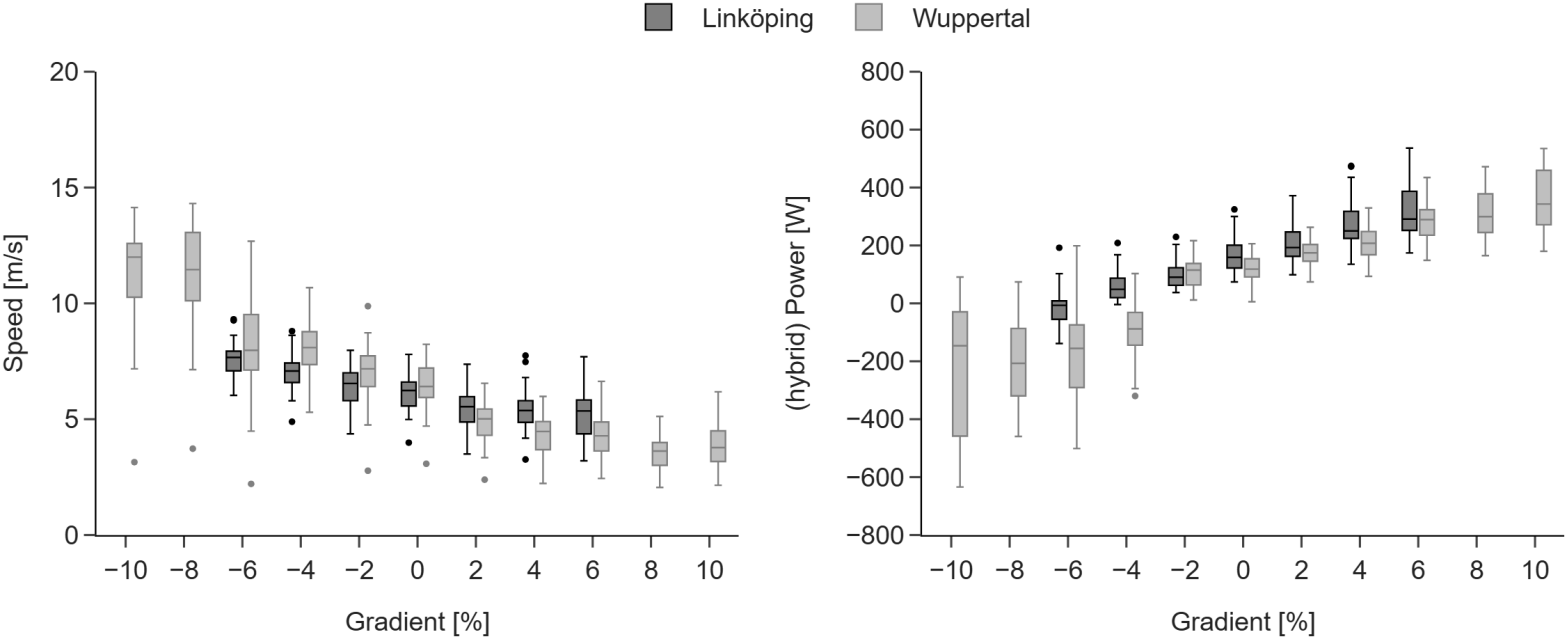
Distributions of speed and (hybrid) power for varying longitudinal gradients. Note: the analysis includes observations from straight segments with low wind speeds.

For simulations with context-based speed distributions, we use empirical speed and power distributions from our dataset (see Fig 1). To support the assumption that bicyclists maintain stable relative positions within speed distributions across gradients, we rank bicyclists within each gradient and find an average standard deviation of 0.1 in their percentile ranks across the population, consistent at both locations. This suggests that a bicyclist who has a high speed preference is likely to stay among the faster bicyclists across different gradients.

The results of the mixed-effects model are summarized in Table 1. In models for both speed and power, the explained variance by fixed effects (marginal *R*^2^) is lower in Linköping, suggesting that intrinsic heterogeneity among bicyclists (e.g., socio-demographics, physical capacity, bicycle properties, personal preferences) explains a larger share of behavioral variation in the less topographically challenging environment; conditional *R*^2^ values reflect the added variability explained by differences among bicyclists. As infrastructure becomes more physically limiting, heterogeneity in individual speeds and powers diminishes.

**Table 1:** Mixed-effects model results.

	Speed model		Power model	
Location	Linköping	Wuppertal	Linköping	Wuppertal
**Fixed effects: coefficients ($\beta_{0},\beta_{x}^{1}$) (mean over the population)**				
Intercept, $\beta_{0}$ [m/s or W]	6.105	6.081	174.077	132.211
Uphill gradient [m/s or W per 1%]	−0.072	−0.190	28.636	25.113
Downhill gradient [m/s or W per 1%]	0.049	0.462	−24.760	−25.030
Elevation gain [1/s or W/m]	−0.179	−0.039	0.867	−1.307
Elevation loss [1/s or W/m]	0.321	0.042	−13.549	−4.046
Is uphill ahead? [m/s or W] {1:True}	0.224	–	12.291	–
Curvature [m^2^/s or Wm]	−5.661	–	−485.178	–
Intersection [m/s or W] {1:True}	−0.480	−0.054	−35.278	−21.147
Wind speed [- or Ws/m]	−0.275	–	15.033	–
**Random effects: intercepts and responses (std. deviation)**				
Bicyclist, *u*_0*j*_ [m/s or W]	0.861	1.289	62.128	38.522
Uphill gradient [m/s or W per 1%]	0.075	0.075	9.695	8.380
Downhill gradient [m/s or W per 1%]	0.061	0.200	10.652	14.605
Elevation gain [1/s or W/m]	0.034	0.047	3.341	1.807
Elevation loss [1/s or W/m]	0.073	0.035	6.523	3.153
Is uphill ahead? [m/s or W] {1:True}	0.121	–	15.330	–
Wind speed [- or Ws/m]	0.112	–	11.186	–
**Model fit**				
R^2^ Marginal	0.339	0.714	0.375	0.739
R^2^ Conditional	0.757	0.918	0.711	0.787

1: $\beta_{x}$ show changes in one unit speed or power per unit change in predictors; p-values<0.05 for all model estimates.

**Fig 2:**
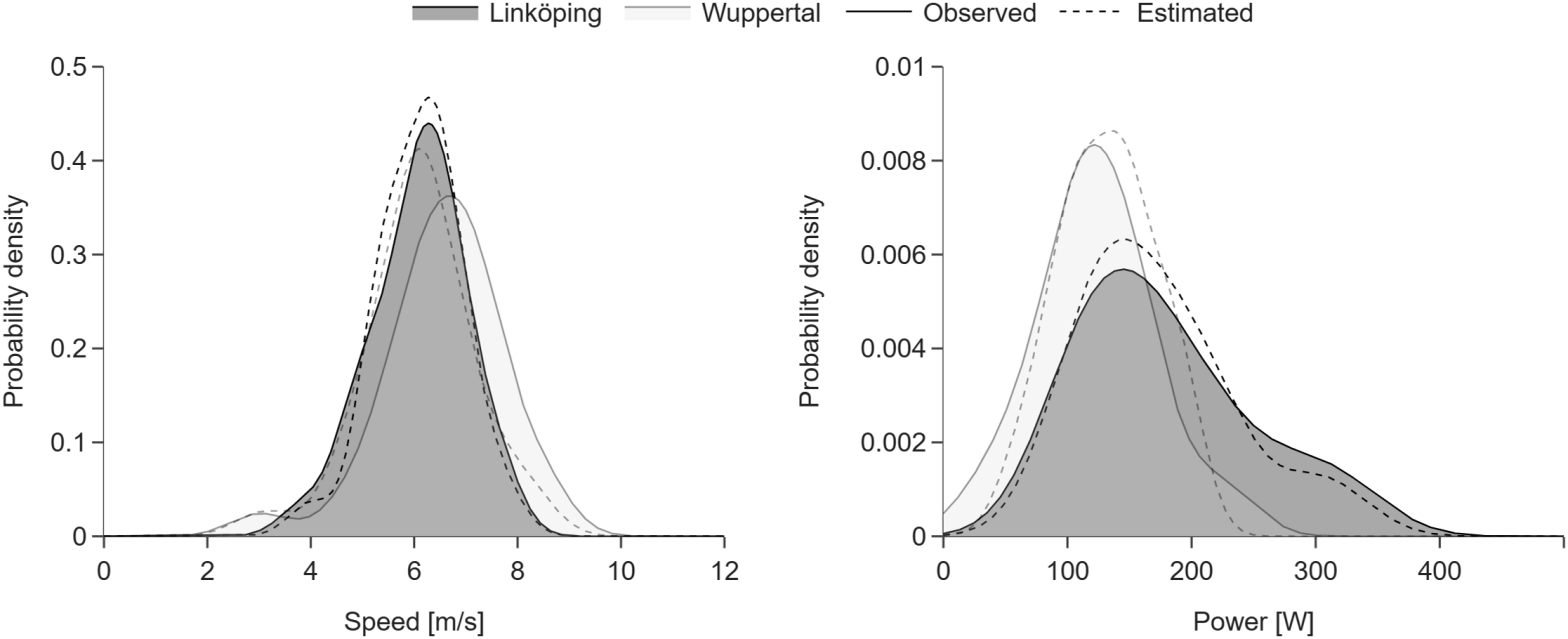
Distributions of desired speed and power, observed versus model estimates (kernel density estimate).

Baseline speeds are similar across sites at approximately 6 m/s (±1 m/s). Baseline powers differ: 174 W (±62 W) in Linköping and 132 W (±39 W) in Wuppertal. Fig 2 shows that baseline distributions for speed and power match the observed distributions, capturing heterogeneity from intrinsic factors such as age, gender, and bicycle type. Kolmogorov–Smirnov tests at the 95% level indicate no significant differences between observed and estimated distributions (p-value>0.063, KS-statistic<0.345).

Much of the observed variation in both speed and power in our datasets is explained by topography, curvature, intersections, and wind speed. Including cumulative elevation gain in our mixed-effects models, alongside local gradients, isolates the instantaneous effect of gradients from the total physical demand of extended hills. This distinction is key to the differences between locations. In Linköping, where hills are shorter, a higher compensatory response to gradients is evident, i.e., bicyclists increase power to mitigate speed losses over brief climbs, reflecting their willingness to exert extra effort for short durations. In contrast, the longer and steeper hills in Wuppertal result in a reduction in power, indicating pacing or fatigue effects. Curves and intersections often occur together, making their effects difficult to disentangle, so each coefficient may partly capture the influence of the other. Model results indicate statistically significant inter-individual variability in responses to local gradients, cumulative elevation gain, tactical anticipatory behavior, and wind speed. Distributions of individual responses are provided in S1 Fig and S2 Fig in the Supporting Information.

In the simulation framework, the mixed-effects models serve two key purposes. The intercept estimates are used to define individual desired speeds and powers. Personalized coefficients for predictors —local gradients, cumulative elevation gain, tactical anticipatory behavior, and wind speed—– are used as simulation parameters to describe variation among bicyclists. Effects from intersections and curvature are modeled to reflect the average impact across the population. Elevation loss is not included as a simulation parameter. Nonetheless, it is necessary to include this variable in the statistical models to isolate its effects from other trip features.

For the physics-based regression speed model, bearing friction coefficients, wheel inertia, and chain efficiency are assumed constant for all bicyclists, with typical values taken from [44], as indicated in Table 2. Air density is computed from the temperature at the time of the experiment. Total mass and wheel radius are personalized for each bicyclist. In Linköping, the resistance parameters $C_{d}A$ and $C_{rr}$ are calibrated individually through coast-down tests (see S1 Appendix for the distributions). Due to the absence of wind speed measurements in Wuppertal, resistance parameters are set to the typical values reported in [58] under similar weather conditions.

**Table 2:** Parameter values used in the bicycle dynamics model.

Parameter	Linköping	Wuppertal
Mass of bicycle–rider unit, *m* [kg]	101.5 (12.9)	98.2 (11.0)
Air density, ρ [kg/m^3^]	1.254 (0.016)	1.165
Drag area, $C_{d}A$ [m^2^]	0.695 (0.133)	0.559
Rolling resistance coefficient, $C_{rr}$ [-]	0.007 (0.002)	0.008
Bearing friction coefficient, $\alpha_{0}$ [N]	0.091	0.091
Bearing friction coefficient, $\alpha_{1}$ [Ns/m]	0.0087	0.0087
Moment of inertia (two wheels), *I* [kg m^2^]	0.14	0.14
Wheel radius, *r* [m]	0.358 (0.0145)	0.36
Chain efficiency factor, η [-]	0.975	0.975
Gravitational acceleration, *g* [m/s^2^]	9.81	9.81

Note: Values represent population means. Values in parentheses indicate the standard deviation across the population of bicyclists.

For all bicyclists, the default maximum values for acceleration and deceleration in all simulation models are set to *a*_max_ = 1.2 m/s^2^ and *b*_max_ = 3 m/s^2^, respectively. These values are based on the Krauss baseline model and aligned with observations, showing no acceleration exceeding these values.

## 5. Results

**Fig 3:**
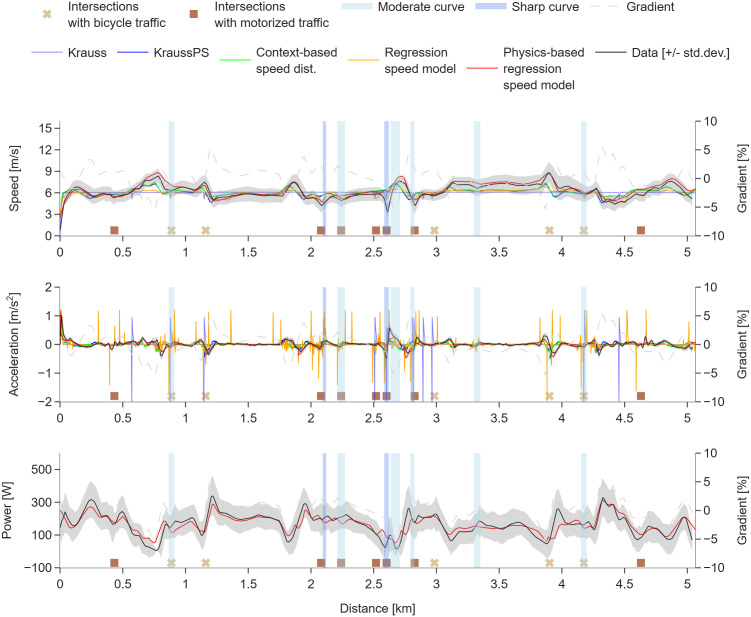
Simulation of free riding speed, acceleration, and power along the Linköping route, mean trajectory over the population.

**Fig 4:**
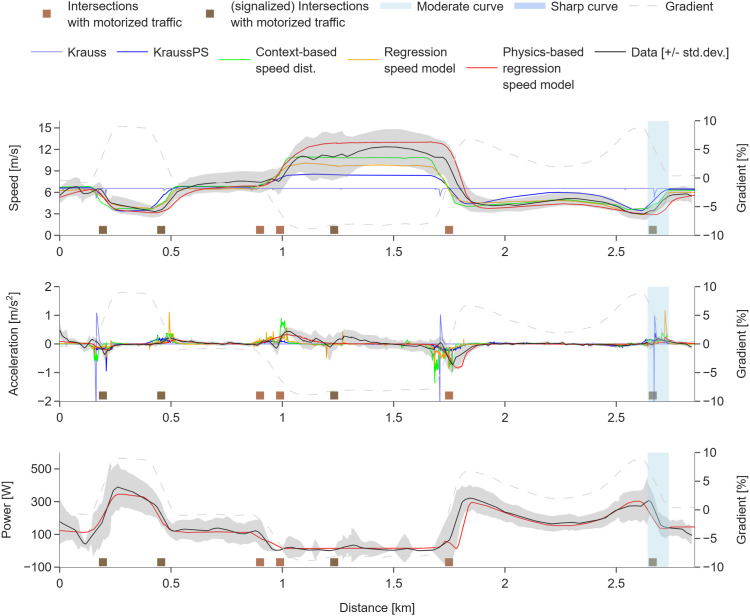
Simulation of free riding speed, acceleration, and power along the Wuppertal route, mean trajectory over the population.

Analysis of the empirical data reveals substantial variation in both speed and power, driven by differences between bicyclists and contextual route conditions, as illustrated in Figs 3 and 4. These findings represent the core dynamics we aim to simulate. Alongside the empirical data, we illustrate the simulated mean speed, mean acceleration, and mean power, over the population and along the route, for each of the simulation models. The default Krauss model illustrates the outcome when the impacts from infrastructure and wind are not explicitly modeled.

All context-sensitive models reproduce the general trends observed in the empirical mean speed profile with varying degrees of fidelity. The Krauss model in SUMO shows occasional peaks of sudden deceleration and acceleration, possibly due to unresolved issues within the simulator. However, these anomalies are not central to our analysis —the key point is how much information is lost when constant desired speeds are assumed. Notably, the physics model most closely aligns with the observed mean speed and acceleration along the route. It also most accurately captures how speed evolves from standstill to the desired cruising speed. Refer to S3 Fig in the Supporting Information for individual simulation results at the start of the trip.

**Fig 5:**
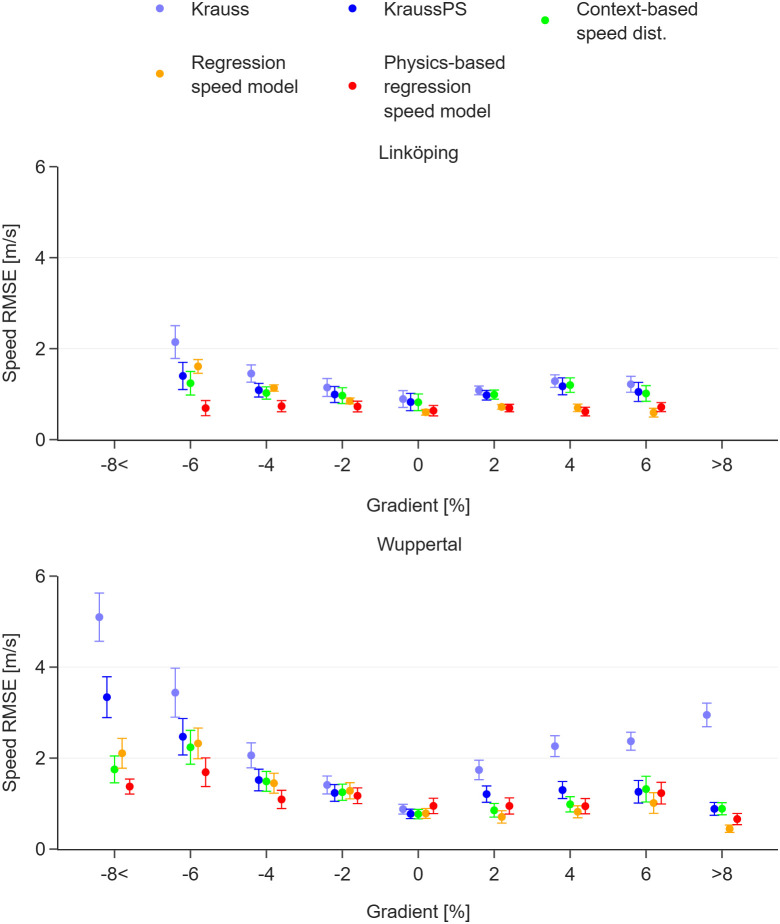
Root Mean Squared Error (RMSE) in speed across longitudinal gradients, confidence interval over the population.

**Fig 6:**
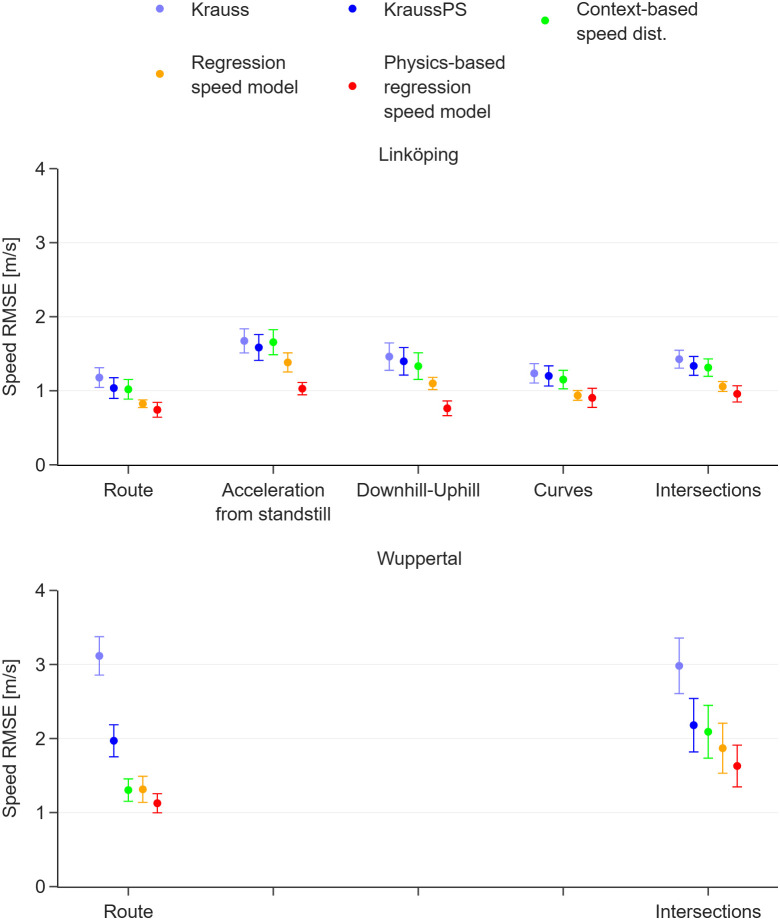
Root Mean Squared Error (RMSE) in speed, confidence interval over the population.

To assess the accuracy of the simulation models, we present summary plots (Figs 5 and 6) comparing the root mean squared error (RMSE) in speed across the participant population. The default Krauss model reflects the level of error expected in simulations that do not account for infrastructure or environmental variation, serving as a baseline for comparison. In contrast, the proposed models capture the observed variability, generally demonstrating lower RMSE values in speed.

The results show clear differences in speed RMSE between models and across gradients and segment types. For downhills, RMSE in speeds significantly increase with gradient in the Krauss models, while context-based speed distributions and the regression speed model reach a peak error at 6 percent gradients; the physics-based regression model shows similar errors across both uphill and downhill gradients. For uphill segments, the default Krauss model also results in rising RMSE with gradient, whereas the remaining models maintain relatively constant RMSE. Note that observations at the steepest gradients are likely influenced by a more limited sample size. RMSE differences between populations likely reflect stronger correlations between local gradients and elevation gain (or loss) in Wuppertal than in Linköping, combined with differing wind conditions between sites.

As the baseline Krauss model shows, omitting context dependency in free riding can result in larger errors in speed variability on uphills and downhills, and in deceleration on curves or at intersections, than our most accurate methods produce. For example, speed RMSE for the whole route is approximately twice as high in Linköping and three times higher in Wuppertal. The KraussPS model improves the simulation of gradient impacts by capturing trends in speed changes, —in specific contexts yielding an RMSE comparable to that of context-based speed distributions— but may systematically overestimate speeds on uphills and underestimate speeds on downhills.

## 6. Discussion

Free riding dynamics exhibit high heterogeneity across participants and show strong dependence on infrastructure and wind speed. Capturing this context dependency is crucial for realistic microscopic simulation of bicycle traffic. In both study sites, the proposed simulation models generally outperform existing SUMO benchmark models in their ability to replicate free riding speeds. Substantial speed errors in simulation analysis may lead to misinterpretations of traffic performance metrics, such as delay. A physics-based model has a greater fidelity and facilitates the simulation of tactical behavior to cope with uphills, and the computation of energy expenditure; power-based frameworks are suitable for examining energy costs and, e.g., studying how exertion influences route choice or willingness to use a bicycle. However, the choice of a suitable method depends on the specific objectives and requirements of the traffic simulation task. The proposed methods differ in accuracy, complexity, and data demands, which enables practitioners to select an approach that best aligns with available data, computational resources, and the level of detail required to represent heterogeneous and context-dependent bicyclist behavior.

Each proposed simulation model offers distinct strengths and limitations. Context-based speed distributions are simple to implement and ensure realistic speeds across varying gradients. However, they cannot capture tactical decisions such as coasting or boosting, and are tied to the wind conditions during data collection. They also require empirical distributions for each gradient bin, making extrapolation to unobserved gradients troublesome. Regression-based models can integrate multiple contextual predictors and adjust speeds dynamically, but their linear and additive assumptions may oversimplify nonlinear behaviors, such as braking and aerodynamic resistance. The regression speed model, in particular, omits inertial and physics-based constraints, which can lead to noisy acceleration estimates. In contrast, the physics-based model derives motion from first principles, updating kinetic energy to generate realistic changes in acceleration, and producing the lowest errors in speed. It also supports the inclusion of tactical behavior related to physical effort, as indicated by the lowest RMSE in downhill-uphill transition where boosting strategies may occur. However, it is more complex and sensitive to calibrated resistance parameters.

The mixed-effects modeling framework captures the heterogeneous and context-dependent dynamics of free riding, making it suitable for integration into traffic simulations. While gender has been identified as a factor influencing speed and power [11], there is insufficient evidence to suggest that men and women respond differently to the examined trip features; such variation by gender is instead captured within the estimated baseline speeds and powers. Furthermore, elevation loss is excluded from the simulation since accumulated effects on downhill segments are often disrupted by stops or intersections, as seen on the Wuppertal route. In contrast, the physiological impact of sustained climbing is more likely to persist even after interruptions, justifying the inclusion of elevation gain as a predictor. Overall, the mixed‐effects models offer a robust basis for simulation by explicitly modeling heterogeneous and context-dependent behavior.

Since the context-based speed distribution model involves minimal computation per time step, it is the most straightforward to integrate into simulations. The regression speed model adds modest complexity through the evaluation of a linear equation at each time step, though its additive structure may produce noisy acceleration estimates that could require additional smoothing. The physics-based model involves numerical integration of bicycle dynamics equations, yet since these equations are closed-form, the computational overhead per time step remains limited. All three models are implemented as single-bicyclist simulations, and their relative computational cost in congested traffic scenarios, where interaction models add further complexity, was not evaluated. Future research should also assess the sensitivity of model outputs such as travel time, speed, and delay to individual parameter choices, to identify opportunities for model simplification without meaningful loss of accuracy.

The observed variability in free riding is expected to influence interactions between bicyclists. While not directly measured in our dataset, such variability is likely to influence overtaking and following regimes, gap‐acceptance decisions, or acceleration profiles. For example, speed differentials during overtaking may depend on wind conditions, horizontal and vertical alignment, individual sensitivity to these features, and tactics for managing physical effort. Steeper hills and stronger winds would also amplify differences between stronger and weaker responses, ultimately influencing overtaking length and duration, and potentially willingness to overtake. Wind conditions may similarly induce tactical following behavior to reduce air resistance. At intersections, heterogeneous approach speeds are likely to alter gap-acceptance thresholds, while acceleration from standstill is further influenced by gradients and wind conditions, affecting queue discharge rates and conflict risk.

Because interactions between bicyclists may be strongly shaped by free riding dynamics, broader implications for bicycle traffic analysis could follow. For instance, fundamental flow relationships on uphill segments likely differ from those on flat terrain: steeper gradients reduce free speeds and compress speed heterogeneity within the population, altering the conditions under which interactions occur. Accounting for this is relevant for representing actual traffic performance where topography varies significantly. By including context-dependent free riding in simulation models, bicycle flow characteristics can be understood as outcomes of the interplay between unconstrained speed choices and interaction mechanisms, enabling more reliable predictions of how traffic performance varies with infrastructure or weather, and reducing the risk of conflating delays caused by traffic with those arising from changes in speed choice.

Since commonly used microscopic simulation frameworks rely on a fixed desired speed, replacing it with a context-sensitive speed estimate provides a flexible mechanism for incorporating free riding dynamics. Although in this paper we examine the approach within the Krauss car-following model in SUMO, the concept of computing a context-dependent targeted speed can be transferable to other simulation frameworks. An alternative approach within the examined SUMO models is to re-parameterize the maximum acceleration and deceleration values for different contexts, as these currently are represented by fixed values. For example, maximum acceleration likely varies between accelerating from a standstill, descending steep downhills, or initiating overtaking. However, adjusting the targeted speed directly, as proposed in this paper, offers a more straightforward path, as it requires a single context-dependent estimate per time step rather than re-parameterizing multiple kinematic constraints across different situations.

Due to the limited sample size, all available data are used for model calibration, leaving no independent dataset for validation. Consequently, the resulting parameter values reflect the characteristics and behaviors observed within our sample. These calibrated parameters are not intended to represent the broader population of bicyclists or the full diversity present in real-world bicycle traffic. Findings reflect the behavior of a subset of bicyclists: frequent commuter bicyclists using non-electric bicycles. The true heterogeneity in broader bicycle traffic is expected to be even greater. Expanding data collection to more diverse populations and contexts is essential to develop validated and generalizable free riding models. Furthermore, our single-bicyclist simulation approach facilitates direct comparison between simulated and observed trajectories but also limits validation to isolated behavior, i.e., additional infrastructure and weather effects that are triggered or intensified by interactions with other road users are not captured.

## 7. Conclusions

Three microscopic models for the simulation of free riding speed dynamics are formulated, implemented in SUMO, and evaluated against empirical trajectory data from two distinct urban environments. In both study sites, the proposed simulation models generally outperform existing SUMO benchmark models in their ability to replicate free riding speeds, particularly with regards to simulating the impact of topography on bicycling. Furthermore, the regression-based models offer the possibility to include several more contextual features, for example, curvature or wind, improving simulation accuracy in speed choices. The physics-based model offers the highest accuracy and richest behavioral representation, while the context-based speed distribution and regression-based speed model provide a competitive and more readily deployable alternative. These approaches together could cover a practical range of modeling needs from detailed traffic analysis to larger-scale simulations.

While the proposed mixed-effects models fairly reproduce speed evolution from standstill to cruising speed (S3 Fig), acceleration phases at trip start —observed under controlled experimental conditions—– may underestimate realistic values from standstill. The 1 Hz sampling rate used in this study is insufficient to determine whether a dedicated acceleration mechanism to reach a desired speed is needed, such as modeling additional power boosts during recovery from low speeds, supported by trajectory data at high sampling rates (>1 Hz) for precise speed evolution.

Future research should evaluate the relative importance of each predictor in the simulation of free riding and explore opportunities for model simplification, e.g., by identifying which parameters can be treated as uniform across bicyclists (or types of bicyclists) and which require individual-specific calibration. Further research should investigate tactical responses to wind, and address speed dynamics in curves and at intersections in greater detail. Efforts in data collection should differentiate the effects of curvature and intersections, allowing for more precise attribution of their influence on speed and power changes. More robust validation of free riding models is needed, incorporating data from a more diverse bicyclist population, trip contexts, and bicycle types. Such work will help develop representative distributions for model parameters and examine how free riding models operate within established frameworks for interactions with other road users, including the evaluation of whether advanced free riding models yield meaningful improvements in the simulation of congested bicycle traffic scenarios, and whether the added accuracy justifies the increased model complexity.

Both the wide heterogeneity in speed choices among bicyclists and their strong dependency on infrastructure and environmental context are meaningful. Accurate simulation of speed choices can thereby enable a more reliable assessment of bicycle traffic performance. Capturing realistic variations in speed and physical effort can inform decisions in the design and evaluation of bicycling facilities, route planning, and policies that better accommodate the diverse preferences and capabilities of bicyclists.

## Supporting information

S1 Checklist Inclusivity in global research. (PDF)

S1 Appendix Calibration of bicycle resistance parameters. (PDF)

S1 FigDistributions of individual responses to trip features (kernel density estimate).Random effects in the mixed-effects models for speed. Note: The distributions represent deviations from the average effect at the population level, $\beta_{x}$.(TIFF)

S2 FigDistributions of individual responses to trip features (kernel density estimate).Random effects in the mixed-effects models for power. Note: The distributions represent deviations from the average effect at the population level, $\beta_{x}$.(TIFF)

S3 Fig Simulation from standstill to a desired speed. Start of the trip, for all bicyclists in Linköping.
(TIFF)
