# Supplementary material for: Microscopic simulation of free riding speed dynamics in bicycle traffic: Modeling heterogeneous context-dependent effects
Source: PLoS One. 2026 Jun 26;21(6):e0351469. doi: 10.1371/journal.pone.0351469 (PMC13309020; doi:10.1371/journal.pone.0351469)
Supplement: S1 Appendix — (PDF) [file pone.0351469.s002.pdf]

S1 Appendix. Calibration of bicycle resistance parameters.

To estimate personalized bicycle resistance parameters, air drag coefficient ($C_d A$) and rolling friction coefficient (C_{rr}), we carry out outdoor coast-down tests for each participant following the method outlined in [1]. We perform the test on a flat and straight 300-meter-long bidirectional bicycle path segment, with a road sign positioned in the middle of the segment; this segment is not part of the designated route used for simulation. Participants are instructed to accelerate to a comfortable speed. Once they pass the road sign, participants start coasting —without braking— until a full stop. Each participant completes four runs on the same segment: two runs in each direction. A single coast-down test takes, on average, 1 minute to perform. For each participant j, we

1. identify coast-down riding in trajectory for calibration, i.e., when bicyclists stop pedaling and until speed drops to 2 m/s; measurement noise increases at very low speeds.

2. determine the optimal parameters $C_d A_j$, $C_{rr,j}$, and $v_{0,j}$ by minimizing the difference between the observed and predicted distances traveled as:

$$\sum_i \left[x_j(t_i) - \hat{x_j}(t_i)\right]^2 , \tag{1}$$

   in which x_j and $\hat{x_j}$ are the observed and predicted distances traveled, respectively, at time t_i. To solve the optimization problem, we apply the differential evolution algorithm considering parameter boundaries [0.001, 0.02], [0.2, 1.2], [2 m/s, 9 m/s] for $C_d A_j$, $C_{rr,j}$, and $v_{0,j}$, respectively. A time-discrete simulation algorithm is implemented to compute changes in kinetic energy using a bicycle dynamics model [2].

3. define the optimal $C_d A_j$ and $C_{rr,j}$ for each participant as the average across all conducted coast-down tests.

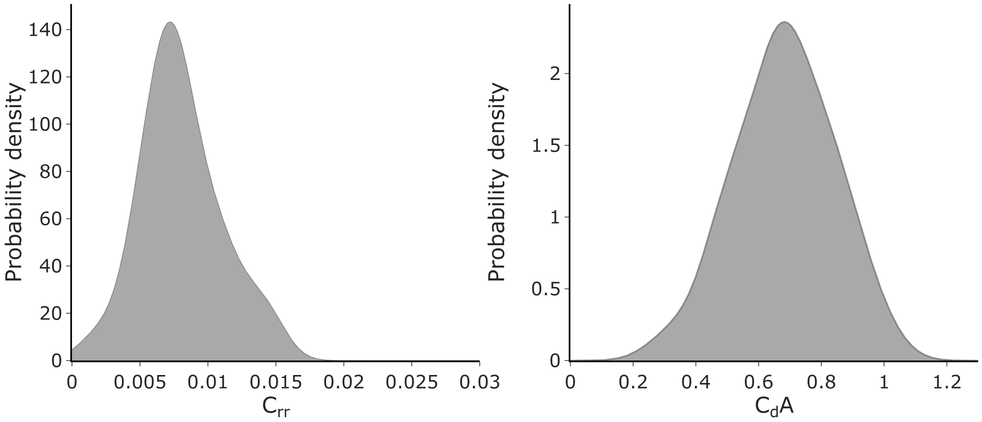

Distribution of bicycle resistance parameters, aerodynamics drag $C_d A$ and rolling friction C_{rr} coefficients, over the population of participants in the Linköping experiment (kernel density estimate).

References

1. Tengattini S, Bigazzi A. Validation of an Outdoor Coast-Down Test to Measure Bicycle Resistance Parameters. Journal of Transportation Engineering, Part A: Systems. 2018;144(7):04018031. doi:10.1061/JTEPBS.0000152.

2. Martin JC, Milliken DL, Cobb JE, McFadden KL, Coggan AR. Validation of a Mathematical Model for Road Cycling Power. Journal of Applied Biomechanics. 1998;14:276-91.
