# Supplementary figures and images for: Microscopic simulation of free riding speed dynamics in bicycle traffic: Modeling heterogeneous context-dependent effects
Source: PLoS One. 2026 Jun 26;21(6):e0351469. doi: 10.1371/journal.pone.0351469 (PMC13309020; doi:10.1371/journal.pone.0351469)

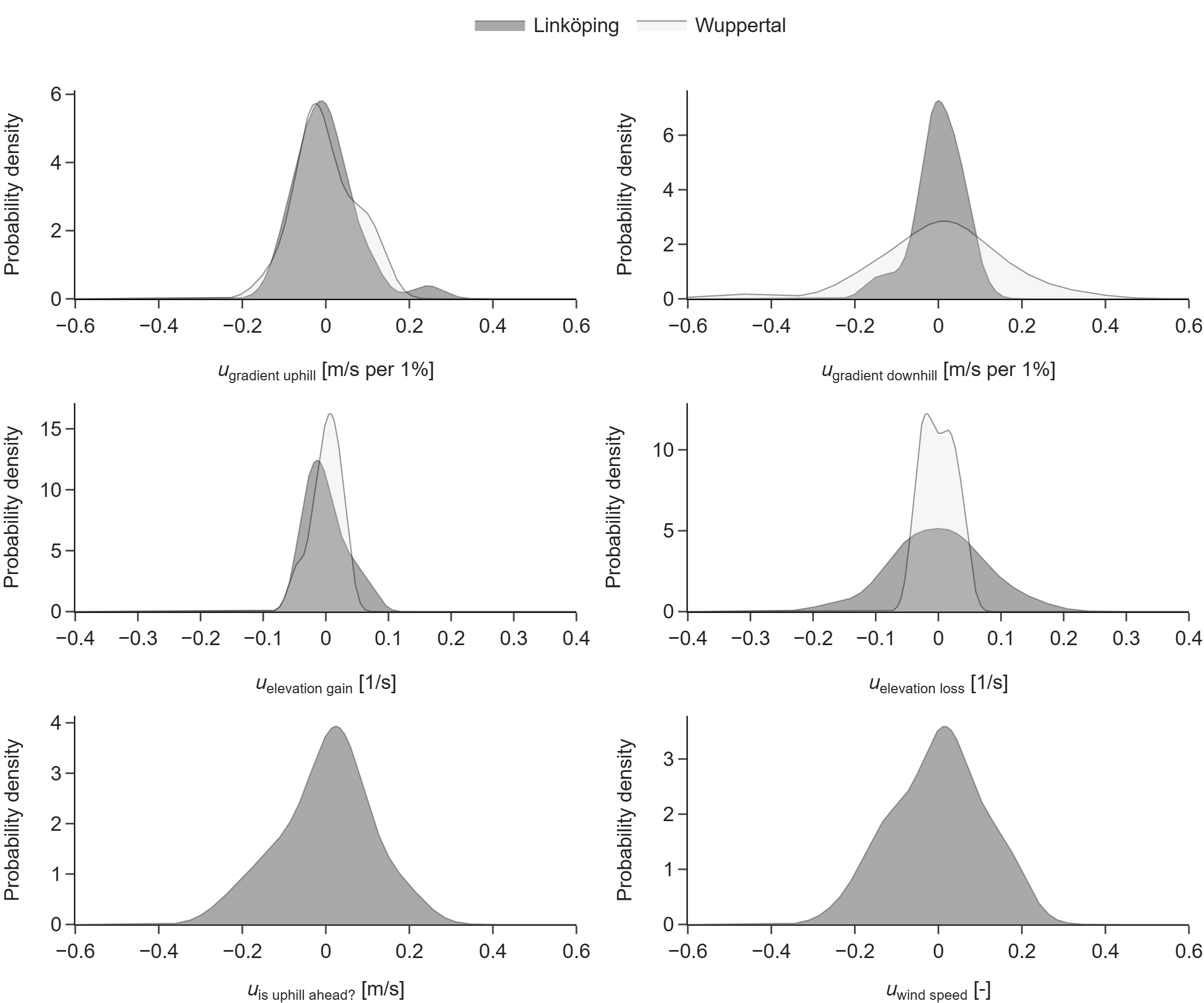

Supplement: S1 Fig — Random effects in the mixed-effects models for speed. Note: The distributions represent deviations from the average effect at the population level, βx. (TIFF) [file pone.0351469.s003.tiff]

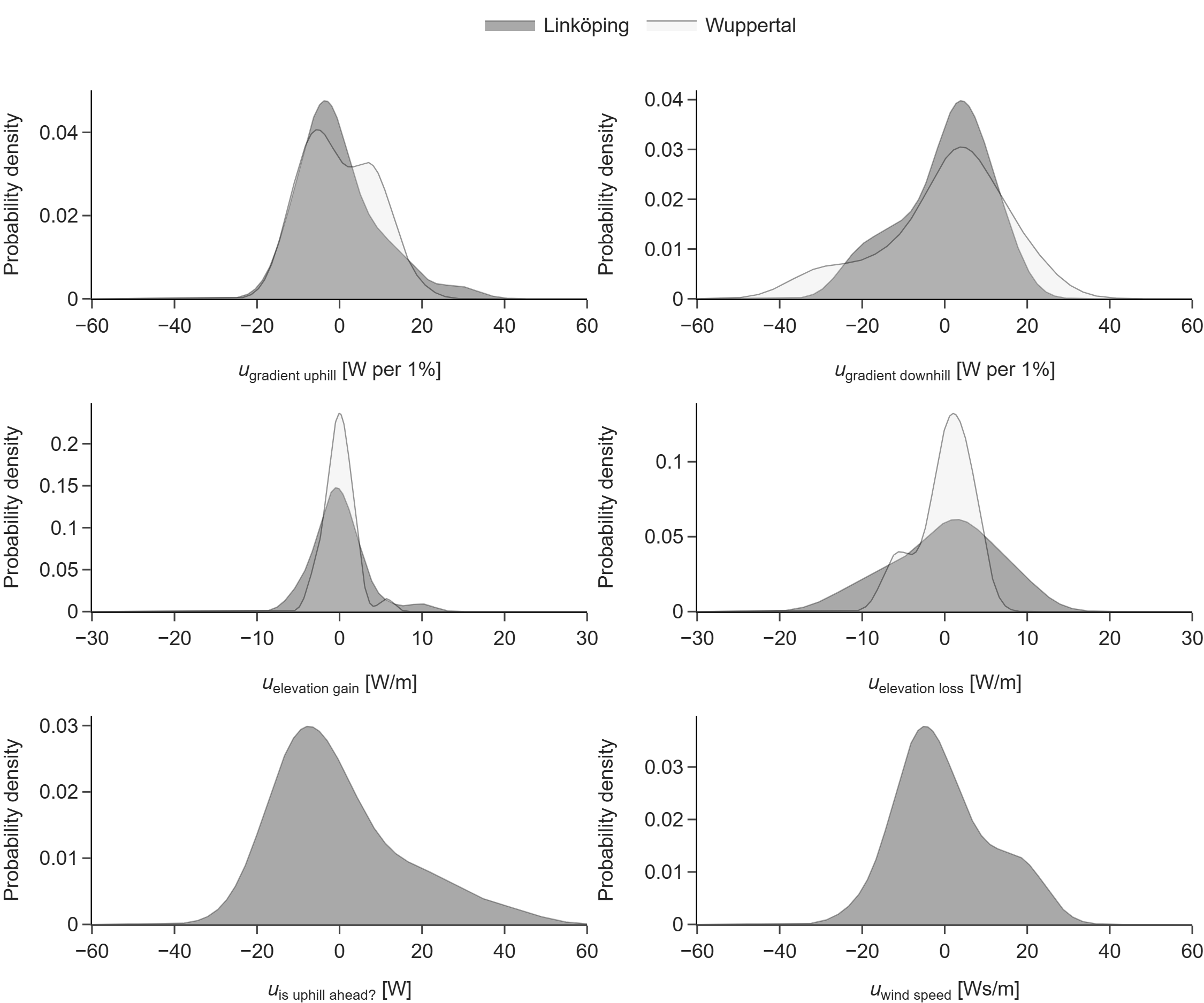

Supplement: S2 Fig — Random effects in the mixed-effects models for power. Note: The distributions represent deviations from the average effect at the population level, βx. (TIFF) [file pone.0351469.s004.tiff]

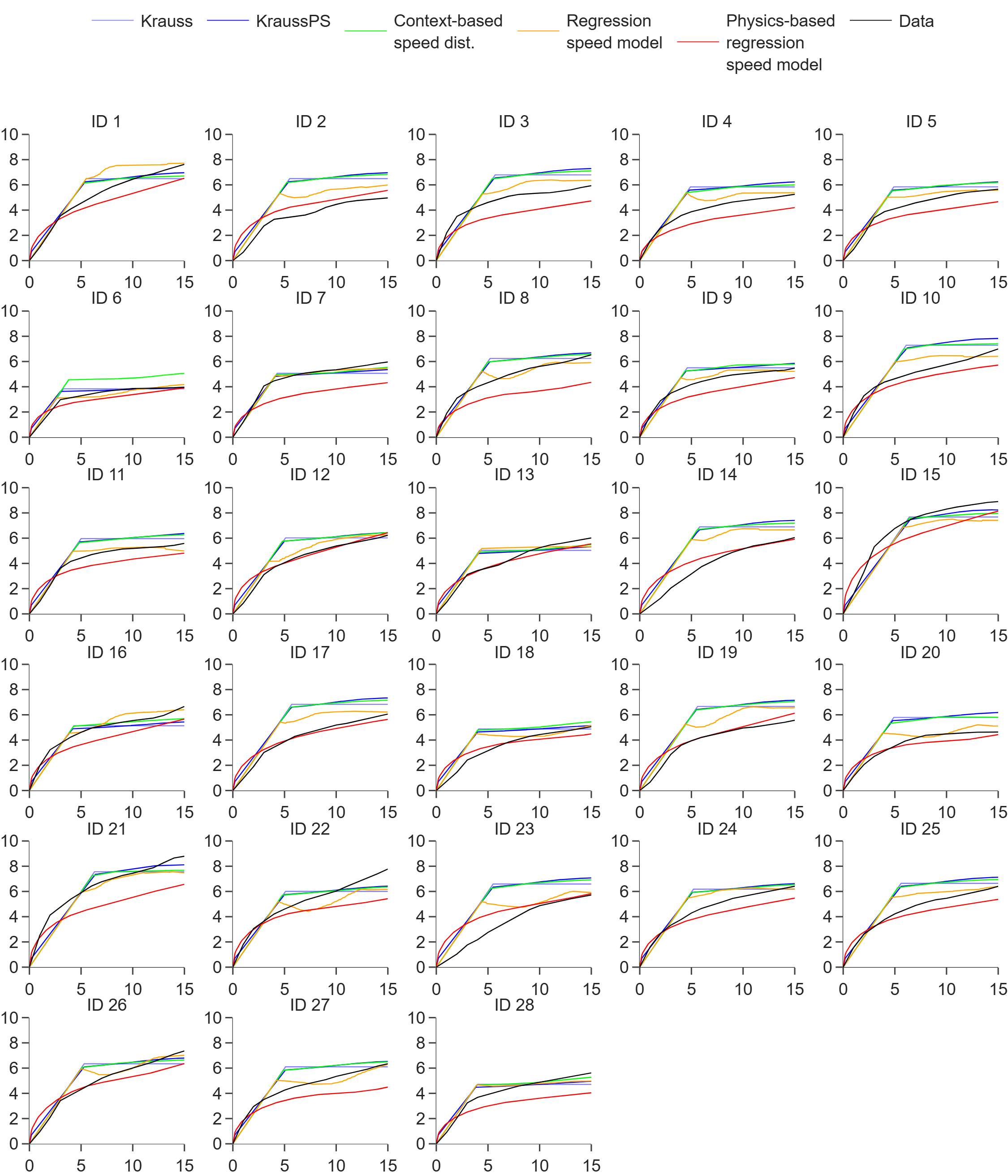

Supplement: S3 Fig — Start of the trip, for all bicyclists in Linköping. (TIFF) [file pone.0351469.s005.tiff]
