# Supplementary material for: Microscopic simulation of free riding speed dynamics in bicycle traffic: Modeling heterogeneous context-dependent effects
Source: PLoS One. 2026 Jun 26;21(6):e0351469. doi: 10.1371/journal.pone.0351469 (PMC13309020; doi:10.1371/journal.pone.0351469)
Supplement: Attachment — Submitted filename: Review_Response_1.pdf [file pone.0351469.s007.pdf]

Response to reviewer's comments

May 15, 2026

We sincerely appreciate and thank the editor and reviewers for their time and valuable feedback on our manuscript. Below, we provide a response to each comment raised, and revisions to the manuscript are highlighted in red. In case of any questions or concerns about the paper, do not hesitate to contact us.

Sincerely,

The authors of paper PONE-D-26-04845

Reviewer 1

Comment 1: The manuscript currently lacks chapter numbering, making it difficult to distinguish between main sections and subsections. Please add appropriate chapter numbers throughout the text.

Response:

We have updated the manuscript template to include chapter numbering throughout the text.

Comment 2: It is recommended that a dedicated ”Research Gaps” subsection be included in Section 2 (Literature Review) to clearly identify the unresolved issues addressed by this study.

Response:

We have added a dedicated “Research gaps” subsection at the end of Section 2 “Literature review” that summarizes key gaps we address in this study.

Comment 3: For the bicycle dynamic model, please provide a summary table listing the parameter values used in the simulations.

Response:

We agree that a summary table would improve clarity and have included Table 2 “Parameter values used in the bicycle dynamics model” in “Section 4 Parametrization of models”.

Comment 4: All algorithmic descriptions should be presented in a formal algorithm format (e.g., with numbered steps and pseudocode). The current presentation is somewhat difficult to follow and would benefit from improved structure and clarity..

Response:

We have rewritten all algorithmic descriptions in formal pseudocode, with explicit Require/Ensure blocks, numbered steps, inputs, and outputs.

Comment 5: The manuscript contains several typographical errors and formatting inconsistencies. A thorough language and technical edit is strongly recommended to enhance clarity, precision, and overall readability.

Response:

We carefully reviewed the manuscript for typographical errors and formatting inconsistencies, and made minor corrections where needed.

Reviewer 2

Comment 6: The proposed work aims to advance microscopic bicycle traffic simulation. However, there is little discussion on “traffic”. While I understand that free-riding differs from collective bicycle riding, a discussion on this topic (also in relation to SUMO and other software) would strengthen the paper (see, for example, DOI: 10.1016/j.jcmr.2024.100022 and DOI: 10.1016/j.trc.2021.103012).

Response:

The role of free riding models in the broader context of bicycle traffic simulation is indeed an important point. In this paper, we aim to argue that free riding behavior could form the behavioral foundation of a comprehensive microscopic bicycle traffic model, where it not only defines the speed dynamics of an unconstrained bicyclist, but also how it could shape interactions between bicyclists. To provide a clearer discussion on this, we have added a paragraph in Section 1 “Introduction”, and expanded Section 6 “Discussion”, previously merged within both “Results” and “Conclusions” Sections, on the role of free riding models in congested traffic.

Comment 7: The models in SUMO include several parameters, and achieving realistic bicycle traffic reproduction, along with proper model calibration for large-scale simulations, can be challenging. It would be interesting to discuss the computational complexity of the proposed model in comparison to the standard SUMO model and in relation to the resulting outputs.

Response:

We have added a paragraph to Section 6 “Discussion” addressing the computational complexity of the proposed models, also motivating also the need for future research for model simplification.

Comment 8: There have been recent advances in bicycle movement modeling, and I suggest that the authors update the literature review. While implementing all possible models is not feasible, a discussion on the trade-offs between complexity (number of parameters), accuracy (whether observations are incorporated), external factors (e.g., age, weight, power), and model types (e.g., cellular automata vs. microscopic models) would be valuable (see, for example, DOI: 10.1016/j.simpat.2024.102986, DOI: 10.1088/0305-4470/37/6/007, and DOI: 10.1016/bs.atpp.2022.06.001). Given the specifications of each software (e.g., sub-lane models, lane-free movement), a short discussion on the varying model requirements would be useful.

Response:

We have extended the “Literature review” Section with a brief overview of commonly used interaction models for bicycle traffic, and how they typically treat the free riding component. We have also added a paragraph to Section 6 “Discussion” that addresses the implications and suitability of adding free riding models to modeling approaches other than the one examined in this paper.

Comment 9: The data used in this study could be highly valuable to the research community for the development, calibration, and validation of bicycle models, which are currently limited in the literature, as the authors note. Do the authors plan to release their dataset as open access? For reproducibility purposes, I recommend providing a public repository with the implementation of the model(s).

Response:

We have added a Data Availability Statement to the manuscript, which includes repositories to the Wuppertal dataset (available at `https://doi.org/10.5281/zenodo.14391230`) and the implementation of the models in our simulations (available at `https://doi.org/10.5281/zenodo.20160109`). Unfortunately, the Linköping dataset cannot be shared publicly due to data privacy constraints.
